# Crush Injury-induced Finger Compartment Syndrome: A Case Report and Literature Review

**DOI:** 10.7759/cureus.7509

**Published:** 2020-04-02

**Authors:** Francisco Schwartz-Fernandes, Emily McDermott, Jared Culp

**Affiliations:** 1 Orthopaedics, University of South Florida Morsani College of Medicine, Tampa, USA; 2 Orthopaedic Surgery, San Antonio Uniformed Services Health Education Consortium, San Antonio, USA; 3 Orthopaedics, Morsani College of Medicine, University of South Florida, Tampa, USA; 4 Emergency Medicine, Morsani College of Medicine, University of South Florida, Tampa, USA

**Keywords:** compartment syndrome, crush injury, orthopedics, finger, digit, injury, fixation

## Abstract

Isolated finger compartment syndrome is an uncommon condition and is not well-documented. It is usually associated with pain, decreased sensation, and intra-compartmental swelling. We present the case of a finger fracture after a crush injury that developed compartment syndrome, which responded well after surgical fixation and midline skin incision for digital decompression.

A 20-year-old male with a history of a 200 lb crush-injury to the left index finger 24 hours prior presented to the emergency department with decreased sensation and range of motion, deformity, increasing pallor, and severe pain. Radiographs demonstrated a middle phalanx fracture of the index finger. Digital decompression of the index finger through a radial approach, along the middle line, and open reduction internal fixation of the middle phalanx improved perfusion almost immediately.

The patient continued to improve at his one-week, 12-week, and six-month follow-up appointments, with a normal neurovascular exam, a capillary refill of less than two seconds, and, ultimately, he was able to make a full composite fist.

Though finger compartment syndrome is uncommon, it should be suspected in cases where the patient demonstrates hallmark clinical signs and symptoms. Compartment syndrome is a clinical diagnosis that requires urgent diagnosis and intervention and must be suspected regardless of the anatomic location of the injury.

## Introduction

In 1881, posttraumatic contractures, known today as complications from compartment syndrome, were first thoroughly described by Volkmann [1]. Rather than being neurological in nature, he stated that the ischemia was resulting in muscle necrosis and contracture. Later, in 1911, Bardenheuer described the process of releasing the internal pressure through a fasciotomy as a prevention of the contractures described by Volkmann [2]. Eventually, Griffiths was able to describe a possible etiology in 1940 and emphasized injury to the artery with a consequential spasm of the vessel as a likely cause [3]. The concept of compartment syndrome was unified in 1975 by Matsen, highlighting that any increased intracompartmental pressure might result in compartment syndrome [4].

Compartment syndrome occurs when the interstitial tissue pressure within a confined space increases to a level at which cellular function is impaired. This increasing interstitial pressure constricts arterial blood flow, limiting tissue perfusion, and leads to progressive tissue ischemia. If untreated, the pressure and subsequent ischemia eventually give rise to tissue necrosis and irreversible loss of function. The outcome of compartment syndrome ultimately depends on the amount of pressure in the compartment, the duration of the pressurization, and the extent of the tissue injury [5].

The compartments of the phalanges have not been well-defined in the literature. However, the pathophysiology of compartment syndrome dictates that any space-occupying anomaly can cause intracompartmental pressure that exceeds capillary perfusion pressure resulting in ischemia and cellular death. The digital neurovascular bundles are confined by the digital cutaneous ligaments, Grayson’s and Cleland’s ligaments, and thus any excessive digital swelling that occurs may constrict the neurovascular bundles in this space and compromise flow to the digit [6-7]. Cleland’s ligament runs dorsal to the digital nerves and vessels while Grayson’s ligament runs volar to these structures [8]. These ligaments act to anchor the skin to deeper structures through the motion of the digit, provide stability, and protect the neurovascular bundle [6-7]. Grossly edematous fingers may require the midline release of dermal and fascial constriction to open this space and restore tissue perfusion, taking care to place the skin incision away from neurovascular bundles.

This report aims to present the case of a young patient with crush-injury induced compartment syndrome of the left index finger with associated symptoms that responded well to fasciotomy.

## Case presentation

A 20-year old male with a history of a 200lb crush-injury to the left index finger 24 hours earlier presented to the emergency department with decreased sensation, deformity, and severe pain of the left index finger that began about four hours prior to his initial presentation.

**Figure 1:**
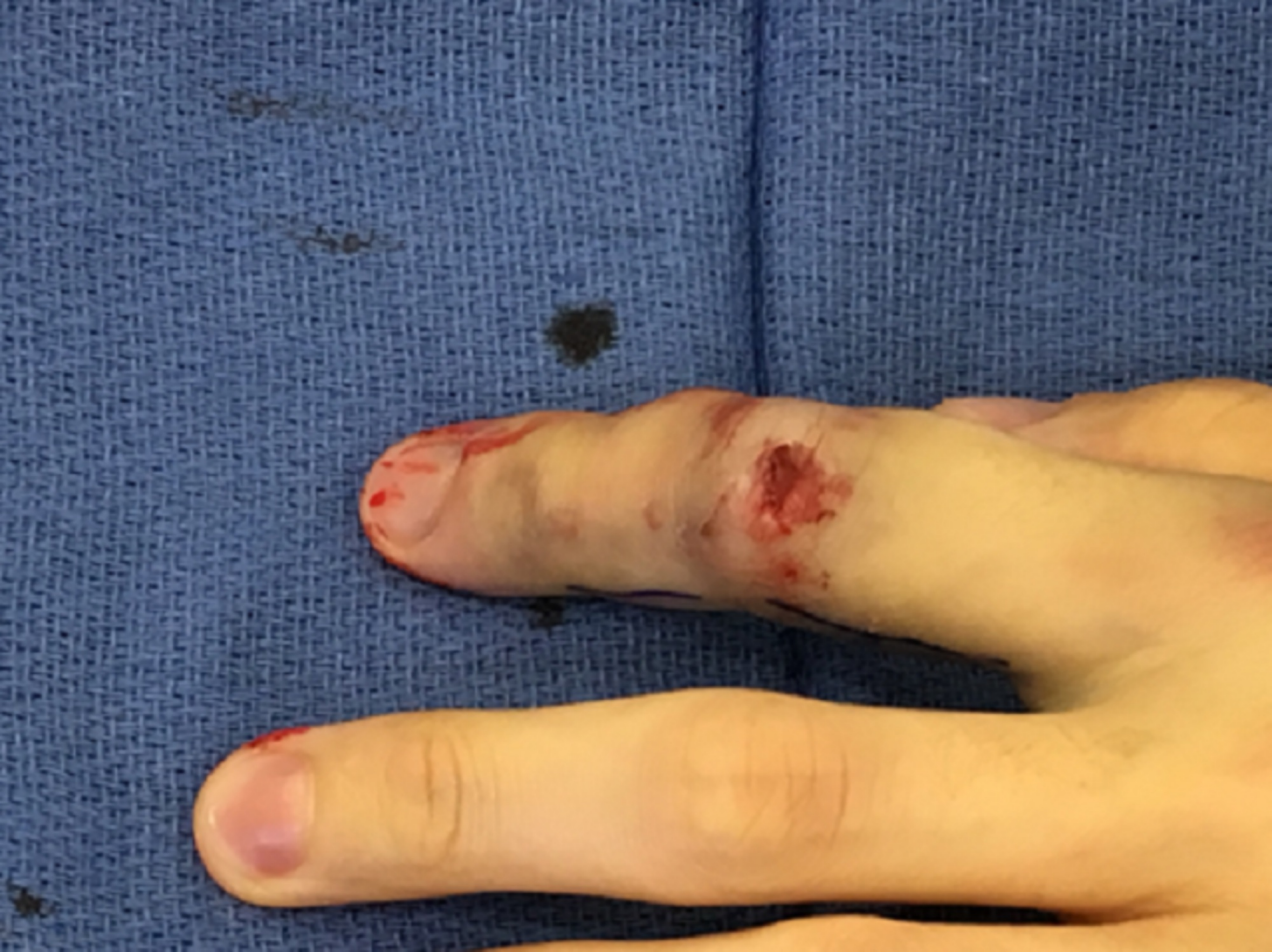
Pretreatment view of the left index finger

The injury was caused by a 200-pound dumbbell that fell on the left index finger. On initial evaluation of the injured finger, the patient reported decreased sensation and increasing pallor along with decreased range of motion (ROM) and lack of improvement in pain with over the counter anti-inflammatories and analgesia. There was obvious deformity of the index finger with a sluggish capillary refill, cyanosis, and severe pain to palpation (Figure 1). It was swollen to twice the size of the contralateral finger. Radiographs demonstrated a fracture of the middle phalanx of the index finger.

**Figure 2:**
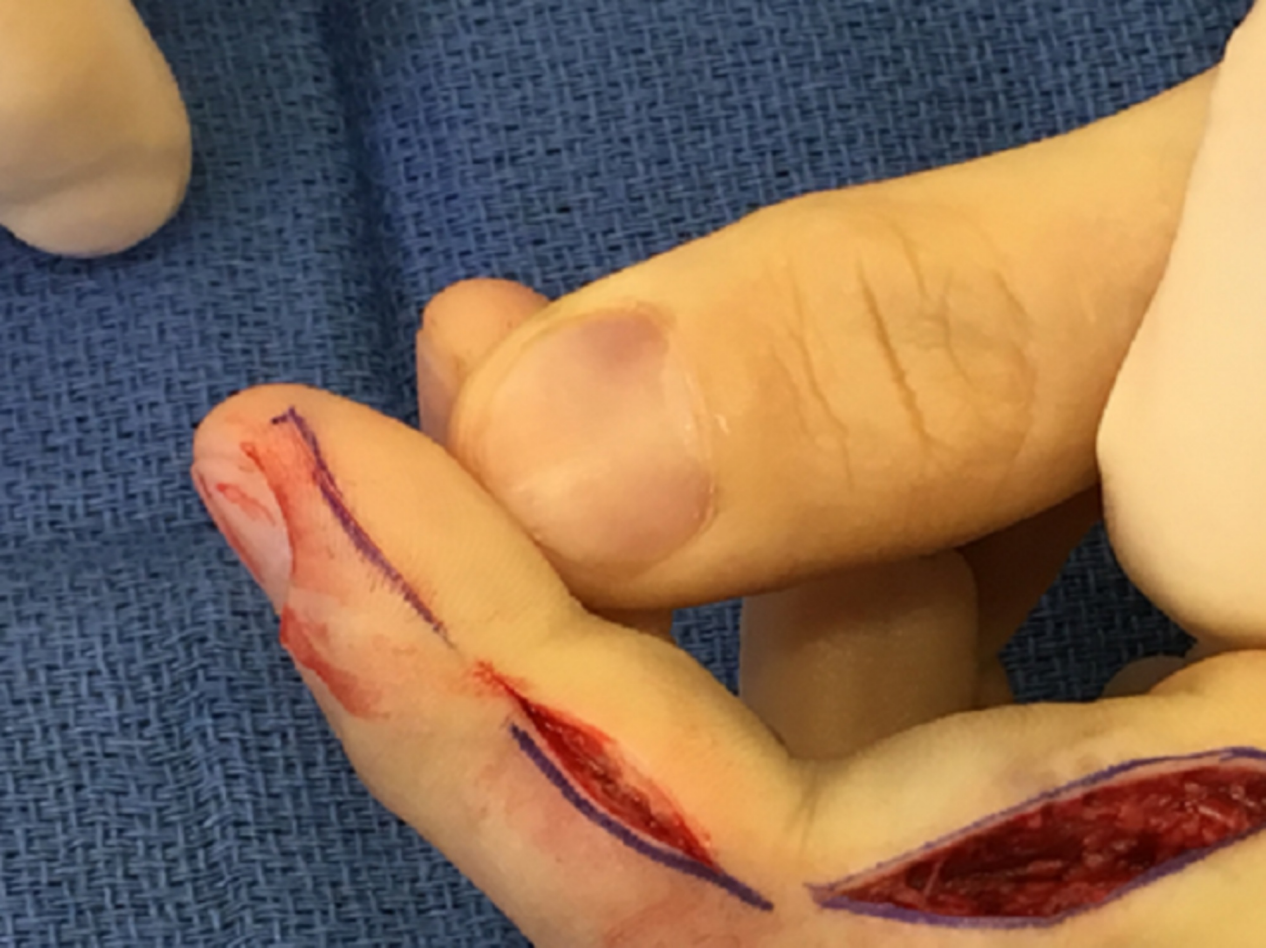
Midline incision of the index finger

**Figure 3:**
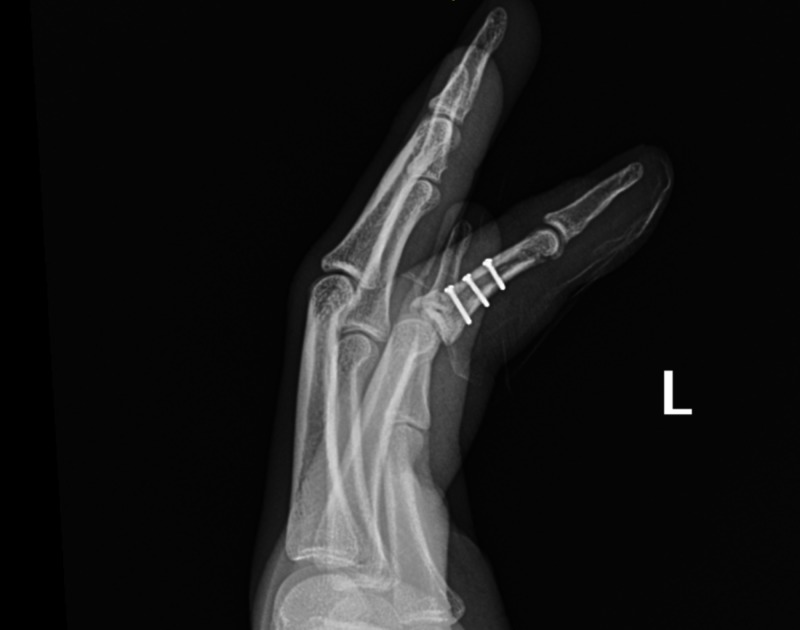
Fixation of the middle phalanx of the index finger

Digital decompression of the index finger through a midline radial approach and open reduction internal fixation of the middle phalanx improved perfusion almost immediately (Figures 2-3).

The patient’s symptoms improved one week later with only moderate pain and improving sensation. Capillary refill improved to less than two seconds. After 12 weeks of hand therapy, range of motion was close to full and the patient’s finger extension was back to baseline. Metacarpophalangeal flexion was 95 degrees, proximal interphalangeal flexion 90 degrees, distal interphalangeal flexion 55 degrees, and he was able to make a near-full composite fist.

At his re-evaluation six months after surgery, the patient was neurovascularly intact with a 2-point discrimination of 7 mm and a capillary refill of 2 seconds, and he was able to make a full composite fist.

## Discussion

The compartments of the phalanges have not been well-defined in the literature and there are few reports of finger compartment syndrome. One of the few studies to address finger compartment syndrome was that by Schnall et al. The study describes an effective operative technique for decompression of the digits in patients with pyogenic flexor tenosynovitis and increased tissue pressures. Their approach included a midlateral skin incision from the distal to proximal phalanx, opting not to release the Grayson’s or Cleland’s ligaments and opening the flexor sheath distally. The sheath was then irrigated with normal saline and Bacitracin. The incision was left open to heal by secondary intention and active movement was started on the first postoperative day. The study found that this operative technique was successful in patients with elevated tissue pressures, yielding promising early results [9]. Avci et al. describe another case of finger compartment syndrome as the result of a forgotten tourniquet. In this case, the clinicians opted for medical therapy after the eventual removal of the tourniquet instead of surgical treatment due to the prolonged ischemia time [10]. Both studies depict increased tissue pressure within the finger as well as some of the hallmark findings of compartment syndrome including severe pain and swelling.

Most commonly, cases of compartment syndrome are related to traumatic injury, especially crushing type mechanisms, which tend to be at particular risk [5]. Even more interesting, patients who develop acute compartment syndrome in the absence of a fracture have been shown to have higher incidences of muscle necrosis when compared to those with fractures [11]. This was not the case in our patient who presented with a middle phalanx fracture of the index finger. There is limited literature describing acute compartment syndrome of the finger or its prevalence.

The diagnosis for compartment syndrome is often clinical, with history and physical exam providing a majority of the information. Our clinical suspicion for compartment syndrome was sufficient to pursue immediate definitive treatment. While the measurement of the interstitial pressure can aid in the diagnosis, it is not required for treatment and there is no consensus about a pressure criterion that defines acute compartment syndrome [5]. Differences between perfusion pressure and tissue pressure can be used clinically as a better marker [12]. In 1940, Griffith described the syndrome with “5 P’s”: painful onset, pallor, paresthesia, paralysis, and, eventually, pulselessness. These 5 Ps continue to be an evaluation method and can be used as clinical hallmarks for diagnosis [3,5,13].

Acute compartment syndrome of any finger is an uncommon finding; however, it is important to consider due to its complications. Hayakawa et al. found in their systematic review of fasciotomies for compartment syndrome that when a fasciotomy was performed within six hours, 88% of the 717 patients assessed had limbs with normal function and without any sensory or motor deficits. When fasciotomy was delayed longer than 12 hours, only 15% of the 94 patients assessed had the aforementioned favorable outcomes. While the cases included in this study did not include finger injuries but, rather, mostly “injuries below the elbow and knee,” it highlights the importance of rapid diagnosis and immediate intervention to avoid sequelae from compartment syndrome [14].

## Conclusions

Clinicians must be highly suspicious of compartment syndrome regardless of the area of injury when presented with the hallmark signs and symptoms. It is an injury that requires urgent diagnosis and intervention to avoid devastating complications. Finger compartment syndrome, though uncommon, should be suspected in crush injuries to the finger resulting in pallor, severe pain, and paresthesia and should undergo urgent digital decompression through a midline incision.

## References

[1] Volkmann R (1881). Die ischaemischen muskellahmungen und kontrak- turen [Article in German]. Centralbl Chir Leipz.

[2] Bardenheuer L (1911). Die entstehung und behandlung der ischämischen muskelkontraktur und gangrän [Article in German]. Dtsch Z Chir.

[3] Griffiths DLL (1940). Volkmann’s ischaemic contracture. Br J Surg.

[4] Matsen FA 3rd (1975). Compartmental syndrome. An unified concept. Clin Orthop Relat Res.

[5] Leversedge FJ, Moore TJ, Peterson BC, Seiler JG III (2011). Compartment syndrome of the upper extremity. J Hand Surg Am.

[6] Chrysopoulo MT, McGrouther DA, Jeschke MG, Kaufman BR (2002). Cleland's ligaments: an anatomic study. Plast Reconstr Surg.

[7] Zwanenburg RL, Werker PMN, McGrouther DA (2014). The anatomy and function of Cleland's ligaments. J Hand Surg Eur Vol.

[8] de-Ary-Pires B, Valdez CF, Shecaira AP, de Ary-Pires R, Ary Pires-Neto M (2007). Cleland's and Grayson's ligaments of the hand: a morphometrical investigation. Clin Anat.

[9] Schnall SB, Vu-Rose T, Holtom PD, Doyle B, Stevanovic M (1996). Tissue pressures in pyogenic flexor tenosynovitis of the finger. Compartment syndrome and its management. J Bone Joint Surg Br.

[10] Avci G, Akan M, Yildirim S, Aköz T (2003). Digital neurovascular compression due to a forgotten tourniquet. Hand Surg.

[11] Hope MJ, McQueen MM (2004). Acute compartment syndrome in the absence of fracture. J Orthop Trauma.

[12] McQueen MM, Gaston P, Court-Brown CM (2000). Acute compartment syndrome. J Bone Joint Surg.

[13] Chopra R, Hayton M, Dunbar PJA (2009). Exercise-induced chronic compartment syndrome of the first dorsal interosseous compartment of the hand: a case report. Hand.

[14] Hayakawa H, Aldington D, Moore A (2009). Acute traumatic compartment syndrome: a systematic review of results of fasciotomy. Trauma.

